# Social Isolation Among Older Adults in the Time of COVID-19: A Gender Perspective

**DOI:** 10.3389/fpubh.2022.840940

**Published:** 2022-06-09

**Authors:** Léna Silberzan, Claude Martin, Nathalie Bajos, EpiCov Study Group, Nathalie Bajos

**Affiliations:** ^1^IRIS, Inserm, Aubervilliers, France; ^2^Arènes (UMR 6051), CNRS, EHESP, Rennes, France; ^3^IRIS, Inserm/EHESS, Aubervilliers, France

**Keywords:** social inequalities, social contacts, COVID-19, gender, older adults

## Abstract

We aimed to analyze inequalities in social isolation among older adults in a time of COVID-19 social restrictions, using a gender perspective. A random population-based survey, including 21,543 older adults (65+) was conducted during and post COVID-19 lockdown in France. Our main outcome was a three-dimension indicator of social isolation based on living conditions, i.e., living alone (i) and not having gone out in the past week (ii), completed by an indicator measuring Internet use i.e., never using the Internet (iii). Logistic regressions were used to identify factors associated with isolation for women and men. Women were more likely to live alone (aOR = 2.72 [2.53; 2.92]), not to have gone out in the past week (aOR = 1.53 [1.39; 1.68]), and not to use the Internet (aOR = 1.30 [1.20; 1.44]). In addition to gender effects, being older, at the bottom of the social hierarchy, and from an ethno-racial minority was also associated with social isolation. Preventive policies should take into account these inequalities when addressing the issue of social isolation among older women and men, so as to enable all social groups to maintain social contacts, and access health information.

## Introduction

Since the beginning of the COVID-19 pandemic, older adults, over-represented among COVID-19 infected people and deaths all around the world (1), have been portrayed as a vulnerable group (2, 3). The epidemiological reality and the biological factors underlying higher mortality among older adults have led to consider them as a homogeneous category. However, studies have shown that aging is a gendered and socially constructed process (4, 5) and that health problems and treatments strongly differ according to social characteristics.

In France, care to older adults was traditionally characterized by a familist approach and has now shifted to a mixed model relying on family and public care (6). As a matter of fact, France now has among the highest shares of older adults living in institutions among developed countries (7). However, as “community care” is scarce in France, people living at home rely before all on informal help (family, neighbors, friends) on a daily basis. During the first lockdown, formal and informal help became limited (8), raising the issue of social isolation among older adults. It reminded the country of the thousands of excess deaths during the August-2003-heatwave in France (9), namely among older adults who did not have access to social contacts during the crisis, because living in places affected by the loss of services and social infrastructure (10).

Social relations have been particularly impacted during the Covid-19 pandemic. Mobility restrictions, as it pertains to lockdown policies, have been put in place in many countries around the world to limit the spread of the epidemic (11, 12). In France, during lockdown (from March 17th up to May 11th 2020), people could only leave their place of residence with an exemption certificate to conduct necessary activities, limiting in-person contacts outside the household to activities such as running necessary errands, imperative family reasons, assisting vulnerable persons, consults and provision of care, medication purchase, individual outdoor exercise within 1 km of one's place of residence and for 1 h. Even after the strict lockdown phase, the government and scientists still appealed to the responsibility of older adults to stay safe and limit in-person contacts. These measures impacted both physical contacts, inside or outside the household, and digital contacts (13, 14), contributing to the 25% increase in older adults feeling isolated in their home or neighborhood compared to the pre-lockdown situation (15), and potentially leading to gender (16), and social (17–19) inequalities in social isolation. Those who maintained high levels of social contacts showed better coping mechanisms during lockdown periods (20), as well as lower risks of depression (21, 22), and frailty (23). In this paper we aim to study social inequalities in social isolation, as defined by Berg and Cassel (24), i.e., the absence of social interactions, contacts, and relationships with family and friends, with neighbors on an individual level, and with “society at large” on a broader level.

Based on a random national population-based survey, we aim to analyze gender and social inequalities in social isolation of adults over 65 years old in France from May 2nd to June 2nd 2020, which included 10 days of strict lockdown, considering access to physical and to digital social contacts. In this study, living alone, having gone outside in the past week and the use of the Internet will be considered as proxies for social contacts.

## Materials and Methods

### Study Design and Participants

The cohort was set-up in April 2020, with the general aim of understanding the main epidemiological, social and behavioral issues related to the COVID-19 epidemic in France (25). The data collection period ran from May 2nd to June 2nd, 2020. In France, strict lockdown expanded from March 17th to May 10th.

### Survey

A random sample of 350,000 people aged 15 and over was drawn from the tax database of the National Institute of Statistics and Economic Studies (INSEE), which covers 96% of the population living in France but excludes people living in institutional settings, and in particular older people living in collectivities. People who belonged to the lowest decile of income were over-represented. All those selected were sent a letter to participate in the survey. A total of 134,391 (38.4%) participated in the survey. Individuals were invited to answer the questionnaire online, or by phone for those who did not have Internet access. Furthermore, a random sample of 10% of people with Internet access was interviewed by phone in order to take into account a method collection effect.

Data collected included socio-demographic characteristics, household size and composition, ethno-racial status, health characteristics and the frequency of Internet use. A total of 25,927 individuals over 65, not living in a residential care facility, responded to the survey. Older adults who carry out an occupational activity were excluded from this study. Indeed, they represented a very specific group when it comes to social isolation, as they might be more likely to have social contacts (namely with colleagues or clients). When restricting the sample to individuals not carrying out an occupational activity and residing in Metropolitan France, the size sample was reduced to 21,543.

We used reweighting and marginal calibrations in the survey and sampling design to correct for non-participation bias among those invited. Weights were calculated using socio-demographics characteristics as covariates to estimate participation probability: sex, age group, employment status (active, inactive), and department, that were available in the original sampling frame.

### Measures

#### Social Variables

We considered the following six variables: age, sex, ethno-racial status (based on migration history), socio-professional category combined with level of formal education (based on current or most recent occupation and education) (*Farmers, self-employed and entrepreneurs/Senior executive professionals/Middle executive professionals/Skilled employees and skilled manual workers/Unskilled employees and unskilled manual workers/Never worked and others*), perceived financial situation (*Very good/Good/Fair/Bad to very bad*) and formal education (defined according to the hierarchical grid of diplomas in France) (*No diploma/Primary education/Vocational secondary/Highschool/Highschool* + *2 to 4 years/Highschool* +*5 or more years*). The ethno-racial status distinguished mainstream population, i.e., persons residing in metropolitan France who are neither immigrants nor native to French Overseas Departments (DOM, i.e., Martinique, Guadeloupe, Reunion Island), nor descendants of immigrant(s) or of DOM native. For the minority population, a distinction was made according to the first (immigrants) and second (descendants of immigrants) generations of immigration, and the country of origin. The term racialized refers to immigrants or descendants of immigrants from the Maghreb, Turkey, Asia and Africa (26).

#### Living Condition Variables

We took into account two variables: that of the household composition (*Living alone/With a partner and with or without children/Other compositions*) and that of the population size of the municipality (*Rural area/*<*50,000 inhabitants/[50,000 – 200,000[inhabitants/*>*200,000 inhabitants/Paris area*).

#### Health Variables

Health variables included drinking habits *(Everyday/Once or several times a week/Once or several times a month/Less often/Never*), perceived health status (*Very good/Good/Fair/Bad/Very bad*) and declared chronic anxiety or depression.

### Outcomes

The main outcome of the study was a three-dimension indicator of social isolation, relying on living conditions and lifestyle (*ie*. respondents who lived alone and respondents who did not go out in the past week), and Internet use (*ie*. respondents who do not use the Internet).

To determine their household composition, participants were asked “Who are the people in this dwelling ie: people who lived in the same dwelling as the respondent at the time of lockdown, including the respondent and the children in shared custody?)”: *Your partner/Your 18 and under children/Your 19 and over children/Your 18 and under grandchildren/Your 19 and over grandchildren/Your 18 and under siblings/Your 19 and over sibling/Your parents/Other members of the family/Other persons (friends, hosts, etc…)*. Results were grouped as follows: *Living alone/With a partner and with or without children/Other compositions*.

To measure how many times respondents had gone out in the past week, they were asked “How many times have you left your home in the last 7 days?”: *Never/Only once/2 to 5 times/6 to 10 times/More than 10 times* Results were grouped as follows: *6 times and over/*/*2 to 5 times*/*Only once*/*Never*.

In addition to the living conditions and lifestyle of the respondents, and with the goal of accessing Internet use, the frequency of Internet use was analyzed. To assess the use of the Internet, participants were asked “In the past 3 months, on average, you used the Internet…”: *Almost every day/Not every day, but at least once a week/Less than once a week/Never/I do not have access to the Internet*. Results were grouped as follows: *Regularly* (Almost every day/Not every day, but at least once a week), *Occasionally* (Less than once a week), *No use of the Internet* (Never, I do not have access to the Internet).

### Statistical Analysis

We first described the distribution of living arrangements and lifestyle by gender and age. Then we studied the social distributions of the main social isolation factors, which are (*i*) living alone and (*ii*) not having gone out in the past week, and never using the Internet (*iii)*. We used logistic regressions by gender and for the whole population to measure relations between socio-demographic characteristics and each of these social isolation items adjusted for socio-demographic indicators, living arrangements and lifestyle and health characteristics. Not having gone out in the past week (ii) was also adjusted for the date of the questionnaire, as the survey was carried out during a period of hard lockdown (02/05–10/05) and a period of easing of lockdown (11/05 and onwards). In addition, we performed the same logistic regressions by household compositoin (*ie*. living alone yes/no), factor which may impact going out and using the Internet.

All analyses were performed with the R software (1.3.959). A *P* < 0.05 was considered statistically significant. All figures shown are gross figures and percentages are weighted. Given the sample size, the observed differences were consistently statistically significant. Therefore, no tests are presented for univariable analyses.

## Results

The higher proportion of women in the sample (56.3% of women, 43.7% of men) reflected the demographic structure of the French population. Half of older adults lived in municipalities with <50,000 inhabitants (50.8%, including 23.2% in rural areas). About one in eight women (12.6%) never worked (vs. 2.5% for men), and 12.3% used to be senior executives (vs. 27.9% for men) (Supplementary Table 1), reflecting the gendered division of the workforce in France. Women were over-represented in primary education levels (33.6 vs. 21.1% for men) and under-represented in the highest education level (3.2 vs. 9.4% for men).

Older women were in poorer perceived health: 57.8% reported being in a “good” or “very good” general health (vs. 60.1% of men), with a stronger difference at age 85 and over (35.9 vs. 45.7%). They also reported chronic anxiety or depression more often (10.1 vs. 3.8%) and a lower alcohol consumption (9.4% of women declared drinking alcohol everyday vs. 28.1% of men).

Gender differences were found regarding social connectedness in the time of COVID-19 (Table 1). Women were more exposed to social isolation than men, whether it be for the fact of living alone (38.5% of women vs. 17.9% of men) or not having gone out in the past week (18.9% of women vs. 11.9% of men). Compared to me, they were also more exposed to not using the Internet (32.1% of women vs. 21.4% of men). These differences were found at all ages (Table 1).

**Table 1:** Characteristics of isolation indicators by gender.

	**Lives alone** **(%)**		**Did not go out in the last 7 days (%)**		**Does not use the Internet (%)**	
	**Women**	**Men**	**Women**	**Men**	**Women**	**Men**
**Variable**						
**Age**						
65–69	27.7	17.6	9.1	6	13.3	9.9
70–74	30.3	16.2	10.9	8.8	18.6	15
75–79	38.9	14.8	16.4	12.1	32.5	22.3
80–84	47.4	16.9	24	15.6	49.1	33.1
85 +	63.4	30.3	48.1	32.6	71.8	55.5
**Formal education**						
No diploma	39.6	16.8	33.2	18.9	63.5	51.9
Primary education	42	19.6	21.7	15.8	41.1	28.6
Vocational secondary	33.8	16.8	14	11	19.6	18.8
High school	38.1	19	11	8	13.5	9.5
High school + 2–4 years	37.7	18.3	10.1	6.9	11	6.4
High school + 5 or more years	31.2	16.7	8.5	8.1	4.5	5.6
**Perceived financial situation**						
Comfortable	31.8	19.6	15.4	9.2	22.6	14.4
Decent	35.6	15.8	17.7	11.5	28.9	18.8
Just enough	43	18.5	20.9	13.5	37.8	28.1
Difficult to impossible without going into debt	50.4	26.8	23.8	14.5	44.4	27.9
**Population size of municipality**						
Rural area	31.7	16.5	21.9	12	34.5	24.4
<50,000 inhabitants	37.8	18.6	18.2	11.3	33.9	22.2
[50,000–200,000] inhabitants	43	15.6	17.6	10.3	30	18.5
>200,000 inhabitants	42.2	19.6	19.4	12.1	31.6	19.8
Paris	41.4	18.3	14.7	14.4	26.5	18.9
**Household composition**						
Living alone	100	100	21.7	11.8	41	28.4
With a partner and with or without children			13.9	11.2	22.8	19
Other compositions			32.4	19.5	44.2	29
**Ethno-racial status**						
Mainstream population	39	17.9	18.2	11.1	31	19.9
Racialized first or second-generation immigrants and DOM descendants	30.7	13.8	28.5	18.6	43.4	35.2
Non-racialized first or second-generation immigrants	38.1	20.1	20.2	13.8	35.9	25.1
**Perceived health status**						
Very good	35.2	15.7	9.1	6.3	18.6	10
Good	35.8	16.9	12.5	8	23.8	18.5
Fair	42.6	19.1	24.9	14.2	42	25.6
Bad to very bad	41.7	22.6	45.2	35.1	59	42.4
**Declared chronic disease or physical limitation**						
Declared at least one	40.2	18.1	23.7	14.5	36.4	23.2
Did not declare any	35.5	17.5	10.1	6.8	24.3	18
**Declared chronic anxiety or depression**						
Declared chronic anxiety or depression	45.6	23.1	28.7	21.9	48.2	33.9
Did not declare chronic anxiety or depression	37.8	17.7	17.8	11.5	30.3	20.9
**Date of questionnaire**						
02/05-10/05	37.7	15.3	22.7	12.9	30.1	17.5
11/05-17/05	38.3	18.6	18.3	12.5	30.5	21.5
18/05-01/06	40	21.1	14	9.7	36.4	27.3
	**Total** ***n*** **(%)**		**Total** ***n*** **(%)**		**Total** ***n*** **(%)**	
	3,507 (38.6)	1,469 (17.9)	1,531 (18.9)	951 (11.9)	2,114 (32.1)	1,298 (21.4)

*Notes: N = 21.543*.

*50.4% of women in a “difficult to impossible without going into debt” perceived financial situation lived alone; 6% of men aged 65 to 69 did not go out in the past 7 days; 32.1% of women and 21.4% of men do not use the Internet*.

All things being equal, women were more likely to live alone than men (aOR = 2.72 [2.53; 2.92]) (Supplementary Table 3). An age gradient was found for women (up to aOR = 5.17 [4.38; 6.11] for 85+ compared to 65–69 years old) but not for men (Table 2). Women with a less comfortable perceived financial situation were more likely to live alone than those in a “comfortable” situation (aOR = 3.85 [3.12; 4.76]). The difference was less marked for men (aOR = 2.24 [1.72; 2.93]). Women with no diploma were less likely to live alone (aOR = 0.74 [0.62; 0.88]), compared to those with a high school level. A similar result was found for men. For women, ethno-racial differences were found as the “racialized 1st or 2nd generation immigrants” group was less likely to live alone than the mainstream population (aOR = 0.70 [0.54; 0.90]). Similar results were found for men.

**Table 2:** Logistic regressions of living alone, not having gone out in the past week and never using the internet, by gender.

	**Lives alone**		**Did not go out in the last 7 days**		**Does not use the internet**	
	**Women**	**Men**	**Women**	**Men**	**Women**	**Men**
	**aOR [95% CI]**	**aOR [95% CI]**	**aOR [95% CI]**	**aOR [95% CI]**	**aOR [95% CI]**	**aOR [95% CI]**
**Age**						
65–69 (ref)	1	1	1	1	1	1
70–74	**1.16 [1.04; 1.29]**	0.87 [0.76; 1.00]	1.18 [1.00; 1.39]	**1.49 [1.22; 1.81]**	**1.59 [1.35; 1.87]**	**1.49 [1.24; 1.79]**
75–79	**1.70 [1.50; 1.93]**	0.90 [0.75; 1.07]	**1.91 [1.59; 2.29]**	**2.11 [1.70; 2.63]**	**3.33 [2.80; 3.97]**	**2.52 [2.06; 3.08]**
80–84	**2.54 [2.19; 2.95]**	1.08 [0.89; 1.32]	**2.59 [2.12; 3.17]**	**3.08 [2.43; 3.90]**	**6.80 [5.64; 8.21]**	**4.66 [3.76; 5.77]**
85 +	**5.17 [4.38; 6.11]**	**2.38 [1.93; 2.93]**	**7.86 [6.41; 9.64]**	**7.29 [5.70; 9.31]**	**16.33 [13.21; 20.18]**	**10.47 [8.25; 13.28]**
**Formal education**						
No diploma	**0.74 [0.62; 0.88]**	**0.69 [0.53; 0.88]**	**2.58 [2.07; 3.23]**	**1.64 [1.24; 2.17]**	**9.84 [7.85; 12.34]**	**11.99 [9.09; 15.82]**
Primary education	0.87 [0.76; 1.00]	0.92 [0.76; 1.12]	**1.61 [1.32; 1.96]**	**1.69 [1.32; 2.16]**	**3.83 [3.13; 4.69]**	**3.93 [3.02; 5.11]**
Vocational secondary	**0.78 [0.68; 0.90]**	**0.78 [0.66; 0.93]**	**1.41 [1.15; 1.74]**	**1.27 [1.00; 1.60]**	**1.88 [1.51; 2.34]**	**3.26 [2.53; 4.19]**
High school (ref)	1	1	1	1	1	1
High school + 2–4 years	1.07 [0.93; 1.23]	0.94 [0.78; 1.13]	0.91 [0.72; 1.15]	0.83 [0.63; 1.10]	0.90 [0.70; 1.16]	0.95 [0.68; 1.32]
High school + 5 or more years	1.03 [0.83; 1.26]	0.91 [0.74; 1.12]	0.86 [0.60; 1.23]	1.02 [0.76; 1.36]	**0.37 [0.22; 0.63]**	**0.63 [0.43; 0.94]**
**Perceived financial situation**						
Comfortable (ref)	1	1	1	1	1	1
Decent	**1.45 [1.28; 1.65]**	0.86 [0.73; 1.00]	0.96 [0.81; 1.15]	1.21 [0.98; 1.49]	1.03 [0.85; 1.24]	1.21 [0.98; 1.50]
Just enough	**2.21 [1.92; 2.54]**	1.18 [0.99; 1.40]	1.06 [0.87; 1.29]	**1.38 [1.10; 1.74]**	**1.35 [1.11; 1.65]**	**1.68 [1.34; 2.11]**
Difficult to impossible without going into debt	**3.85 [3.12; 4.76]**	**2.24 [1.72; 2.93]**	1.04 [0.77; 1.40]	1.41 [0.97; 2.03]	**1.53 [1.15; 2.04]**	**1.51 [1.08; 2.12]**
**Population size of municipality**						
Rural area	**0.61 [0.53; 0.70]**	1.05 [0.86; 1.28]	**1.60 [1.30; 1.96]**	**1.46 [1.14; 1.88]**	**1.34 [1.11; 1.64]**	**1.56 [1.24; 1.97]**
<50,000 inhabitants	**0.81 [0.71; 0.93]**	1.02 [0.84; 1.24]	1.19 [0.97; 1.46]	1.14 [0.88; 1.47]	**1.24 [1.02; 1.50]**	**1.30 [1.03; 1.64]**
[50,000–200,000] inhabitants (ref)	1	1	1	1	1	1
>200,000 inhabitants	0.98 [0.85; 1.12]	1.12 [0.92; 1.37]	1.11 [0.90; 1.37]	1.27 [0.98; 1.65]	1.04 [0.85; 1.27]	1.11 [0.87; 1.42]
Paris	1.02 [0.87; 1.21]	1.15 [0.91; 1.46]	0.82 [0.63; 1.07]	1.19 [0.88; 1.62]	0.89 [0.70; 1.14]	0.89 [0.66; 1.21]
**Household composition**						
Living alone (ref)			1	1	1	1
With a partner and with or without children			1.12 [0.97; 1.29]	1.14 [0.93; 1.41]	**0.78 [0.69; 0.89]**	**0.64 [0.54; 0.77]**
Other compositions			**1.72 [1.41; 2.10]**	**1.81 [1.33; 2.47]**	1.05 [0.86; 1.28]	0.97 [0.73; 1.30]
**Ethno-racial status**						
Mainstream population (ref)	1	1	1	1	1	1
Racialized first or second-generation immigrants and DOM descendants	**0.70 [0.54; 0.90]**	**0.72 [0.53; 0.98]**	**1.96 [1.46; 2.64]**	**1.77 [1.30; 2.41]**	**1.40 [1.04; 1.89]**	**1.49 [1.11; 2.01]**
Non-racialized first or second-generation immigrants	0.95 [0.84; 1.09]	1.11 [0.93; 1.32]	0.95 [0.79; 1.14]	1.21 [0.98; 1.49]	1.05 [0.88; 1.25]	1.13 [0.92; 1.38]
**Perceived health status**						
Very good (ref)	1	1	1	1	1	1
Good	0.90 [0.80; 1.01]	1.13 [0.96; 1.33]	1.17 [0.95; 1.43]	1.08 [0.85; 1.37]	1.15 [0.95; 1.39]	**1.65 [1.30; 2.09]**
Fair	0.96 [0.84; 1.09]	1.17 [0.98; 1.40]	**1.82 [1.48; 2.23]**	**1.67 [1.31; 2.12]**	**1.78 [1.46; 2.16]**	**2.16 [1.70; 2.75]**
Bad to very bad	0.81 [0.66; 1.00]	**1.63 [1.28; 2.09]**	**4.66 [3.60; 6.02]**	**5.53 [4.18; 7.31]**	**2.65 [2.04; 3.45]**	**3.62 [2.70; 4.87]**
**Date of questionnaire**						
02/05-10/05			1	1		
11/05-17/05			**0.79 [0.69; 0.90]**	0.88 [0.74; 1.03]		
18/05-01/06			**0.39 [0.33; 0.46]**	**0.45 [0.37; 0.55]**		

*Notes: N = 21.543, aOR = adjusted odd ratio, significant associations are indicated in bold*.

As regard to having gone out in the past week, data showed that women were more likely than men not to have gone out in the past week than men (aOR = 1.53 [1.39; 1.67]) (Supplementary Table 3). A strong age gradient was found for women (up to aOR = 7.86 [6.41; 9.64] for 85+) (Table 2). A similar age gradient was found for men. A gradient for level of education was noted for women with education levels under the high school level (up to aOR = 2.58 [2.07; 3.23] for women without any diploma). A similar gradient was found for men, although it was less pronounced than for women. Women who belonged to the racialized immigrants group were more likely not to have gone out in the past week than women from the mainstream population (aOR = 1.96 [1.46; 2.64]) (Table 2). A similar result was found for men.

When it comes to not using the Internet in the past 3 months, women were more likely not to use the Internet compared to men (1.30 [1.20; 1.44]) (Supplementary Table 3). Furthermore, an age gradient was found for women and men, but was stronger for women [up to aOR = 16.33 [13.21; 20.18] for 85+ vs. aOR = 10.47 [8.25; 13.28] for men (Table 2)]. Women with lower education levels were more likely not to use the Internet: up to aOR = 9.84 [7.85; 12.34] for respondents without any diploma compared to those with a high school degree (Table 2). A similar trend was found regarding financial situations: aOR = 1.53 [1.15; 2.04] for those in a “difficult to impossible without going into debt” compared to those in a “comfortable” perceived financial situation. Similar trends for education level and perceived financial situation were found for men. Results also showed that the racialized immigrant women were more likely to not use the Internet than women from the mainstream population (aOR = 1.40 [1.04; 1.89]) (Table 2). A similar result was found for men. Women living in a municipality with <50,000 inhabitants were more likely not to use the Internet than those living in a municipality with 50,000–200,000 inhabitants (aOR = 1.34 [1.11; 1.64] and aOR = 1.24 [1.02; 1.50]). Those living with a partner were less likely not to use the Internet (aOR = 0.78 [0.69; 0.89]). These results were also found for men.

Finally, it is worth noting that the relation between the perceived financial situation and not having gone out and not using the Internet, was no longer significant when considering those living alone (Supplementary Table 4). Furthermore, the relation between belonging to the racialized immigrant group was not associated with not having gone out, when considering those living alone.

## Discussion

Our findings provide contextual information on social isolation of older adults during the first national lockdown in France based on a population-based random survey. To question the so-called vulnerability of this population (27), we focused on social variations of specific living arrangements and practices, *ie*., living alone, not having gone outside the home, and not using the Internet. In a Covid-19 context of limited in-person contacts, we found that women were more likely to live alone, not having gone out in the past week and not using the Internet. In addition to gender effects, being older, less educated, in economic precariousness, and belonging to racialized minorities were associated with living alone and not using the Internet.

Among the three indicators that we used to describe and characterize social isolation, living alone was not a consequence of the pandemic, as 97.5% of older adults stayed in their regular place of residence during lockdown (15). The pandemic, and the associated period of strict limited-contacts might have, however, put a dire strain on individuals living alone.

Our results confirmed the importance of demographic and social issues in accounting for the characteristics of older people in France. To begin with, older women lived more often alone as they got older, compared to men of the same age, which refers to the excess male mortality rate, but also to age differences between spouses (28, 29). Secondly, a larger proportion of women than men did not have any diploma and never worked, which reflects the gendered socialization and division of the workforce in France. This accounts for the stronger economic precariousness of older women whether they live alone or not.

Our analysis opened new points of discussion on gender inequalities. Perceived financial status, closely related to the income level, was associated with living alone, especially among those in poorer financial situations. Living alone, as a result of widowhood or divorce has strong financial consequences (30), especially for women. Compared to men, women, living alone or not, were also less likely to have physical contact outside the household by going out. They may be more likely to perceive the pandemic as a serious health issue and therefore to fully agree to comply with restrictive measures, such as limiting contacts outside the household (31, 32). This result may also reflect the long-term socialization process that assigns domestic responsibilities in the household to women. The relation between low education level and lower likelihood to have gone out was stronger among women than among men, possibly referring to the double effect of higher risk perception in low-educated groups and higher protective behaviors, such as limiting social contacts, of women regarding Covid-19 (33). Moreover, women were found to be less likely to use the Internet than men, especially at older ages. A similar result was found in a US study on the Internet use of older adults at the time of COVID-19 (13). This gender gap is likely to refer to a gendered socialization process as women have gained less experience and skills before retirement and therefore have higher barriers toward adopting and using innovative technology in later life (34).

In addition to gender effects, we found marked social differences. The odds of not going out were lower for those living alone, which could relate to the higher frequency of the necessity of going out to conduct necessary activities, such as running errands, when living alone. Moreover, lower levels of education were associated with not having gone out in the past week. Research is scarce on the topic, although we could hypothesize a lower health literacy level (35) and therefore an increased fear of going out. Regarding the use of the Internet, participants with lower levels of education and perceived difficult financial situation were less likely to use it, which is consistent with other studies in the UK on the use of the Internet in later life (36). The Internet was also less likely to be used by participants living in low-populated areas, which are more often lagging behind when it comes to digital infrastructures (37). Finally, the association between lower perceived financial situation and not having gone out and not using the Internet, was significant only for people who do not live alone. As people in lower economic groups are more likely to live in an intergenerational household (38), they might have relied on others to run necessary errands and use the Internet.

Findings also highlight the specific effects added from the geographic origin. Indeed, regardless of gender or social class, racialized 1st or 2nd generation immigrants lived less often alone than the mainstream population. This could be partly explained by late family reunification procedures and by the fundamental supporting role of the family in network ties of immigrants, especially that of the first generation, that lead to intergenerational cohabitation (39). When considering only those who do not live alone, our study found that they went out significantly less than the mainstream population, which could also be partly explained by the fact that their children play an essential part in helping them in their daily lives (39), possibly preventing them from going out. They also had lower levels of Internet use, a possible consequence of a later access to new technologies than the mainstream population (40), and possible barriers to accessing digital health information (41).

This study enabled us to identify categories of older adults who cumulate strong exposure to several social isolation indicators. Women with lower incomes and level of qualification, racialized 1st or 2nd generation immigrants, and people living in rural areas were less likely to go out in the last 7 days and more likely not to use the Internet. Furthermore, a cumulative effect of gender, age and perceived financial situation was observed. Thus, older adults in a precarious financial situation, and before all older women, were more concerned by social isolation, in the sense that they accumulated the likelihood of living alone, not going out, and not using the Internet. We could assume that these groups suffered a “double lockdown” during the first wave of Covid-19 in France (18), suffering the consequences of enforced self-isolation, and the loss of services and social infrastructure.

Our analyses presented some limitations. People in retirement homes were not included in this inquiry, which prevented us from being fully representative of the French population over 65 years old. The indicators used in the study would have benefited from further development. For example, the fact that participants did not go out in the past week does not mean that they were totally deprived of physical contacts from the outside, such as visits from relatives or help from remunerated assistance. Moreover, details on how many contacts the person had when going out would have provided information on the person's social network, even though at the time of the survey, it was strictly recommended by Public Health authorities not to have contacts with older adults.

Finally, our results highlight gender and social inequalities in social isolation, women and especially older women, but also women living in low-populated areas (half of older adults in France), living alone, from low-educated or low-economic groups, or from racialized minorities being more likely cumulate isolation factors. In particular, these groups were less likely to have access to the Internet, and therefore not only to online services and health information, but also to social networks and opportunity to develop them. As women are socially considered the pillar of social contacts and family relationships, this networking capacity may be considered as crucial, in a context where collective togetherness was mainly organized through Internet-based communication networks.

## Data Availability Statement

The data analyzed in this study is subject to the following licenses/restrictions: Data of the study are protected under the protection of health data regulation set by the French National Commission on Informatics and Liberty (Commission Nationale de l'Informatique et des Libertés, CNIL) in line with the European regulations and the Data Protection Act. The data can be available upon reasonable request to the co-principal investigator of the study (nathalie.bajos@inserm.fr). The French law forbids us to provide free access to EPICOV data; access could however be given by the EPICOV steering committee after legal verification of the use of the data. Please, feel free to come back to us should you have any additional questions.

## Ethics Statement

The studies involving human participants were reviewed and approved by the CNIL (French independent administrative authority responsible for data protection), the Comité de protection des personnes (French equivalent of the Research Ethics Committee), and the Comité du Label de la statistique publique. Written informed consent to participate in this study was provided by the participants' legal guardian/next of kin.

## EpiCov Study Group

Nathalie Bajos (co-principal investigator), Josiane Warszawski (co-principal investigator), Guillaume Bagein, Muriel Barlet, François Beck, Emilie Counil, Florence Jusot, Aude Leduc, Nathalie Lydie, Claude Martin, Laurence Meyer, Philippe Raynaud, Alexandra Rouquette, Ariane Pailhé, Nicolas Paliod, Delphine Rahib, Patrick Sillard, Rémy Slama, Alexis Spire.

## Author Contributions

LS: conceptualization, software, formal analysis, and writing-original draft. CM: conceptualization and writing-review & editing. NB: conceptualization, writing-original draft, and supervision. All authors contributed to the article and approved the submitted version.

## Funding

This work was supported by Inserm (Institut National de la Santé et de la Recherche Médicale); the French Ministry for Research; and the DREES (Direction de la recherche, des études, de l'évaluation et des statistiques). The funders facilitated data acquisition but had no role in the design, analysis, interpretation, or writing. This project has received funding from the European Union's Horizon 2020 research and innovation programme under grant agreement No. [101016167], ORCHESTRA (Connecting European Cohorts to Increase Common and Effective Response to SARS-CoV-2 Pandemic). NB has received funding from the European Research Council (ERC) under the European Union's Horizon 2020 research and innovation programme (grant agreement No. [856478]), and from Horizon 2020 European research Council (Gendhi-Synergy grant agreement N° [SGY2019-856478]). The funders had no role in study design, data collection and analysis, decision to publish, or preparation of the manuscript.

## Conflict of Interest

The authors declare that the research was conducted in the absence of any commercial or financial relationships that could be construed as a potential conflict of interest.

## Publisher's Note

All claims expressed in this article are solely those of the authors and do not necessarily represent those of their affiliated organizations, or those of the publisher, the editors and the reviewers. Any product that may be evaluated in this article, or claim that may be made by its manufacturer, is not guaranteed or endorsed by the publisher.

## References

[1] Verity R Okell LC Dorigatti I Winskill P Whittaker C Imai N. Estimates of the severity of coronavirus disease 2019: a model-based analysis. Lancet Infect Dis. (2020) 20:669–77. 10.1016/S1473-3099(20)30243-7 32240634 PMC7158570

[2] Ayalon L. There is nothing new under the sun: ageism and intergenerational tension in the age of the COVID-19 outbreak. Int Psychogeriatr. (2020) 32:1221–4. 10.1017/S1041610220000575 32284078 PMC7184144

[3] Heid AR Cartwright F Wilson-Genderson M Pruchno R. Challenges experienced by older people during the initial months of the COVID-19 pandemic. Gerontologist. (2021) 61:48–58. 10.1093/geront/gnaa138 32955079 PMC7543473

[4] Bengtson VL Burgess EO Parrott TM. Theory, explanation, and a third generation of theoretical development in social gerontology. J Gerontol Series B: Psychol Sci Soc Sci. (1997) 52B:S72–88. 10.1093/geronb/52B.2.S72 9060987

[5] Perkinson MA Solimeo SL. Aging in cultural context and as narrative process: conceptual foundations of the anthropology of aging as reflected in the works of margaret clark and sharon Kaufman. Gerontologist. (2014) 54:101–7. 10.1093/geront/gnt128 24270212

[6] Le Bihan B Da Roit B Sopadzhiyan A. The turn to optional familialism through the market: long-term care, cash-for-care, and caregiving policies in Europe. Soc Policy Admin. (2019) 53:579–95. 10.1111/spol.12505

[7] OECD. Workforce and Safety in Long-Term Care During the COVID-19 Pandemic - OECD. (2020). Available online at: https://read.oecd-ilibrary.org/view/?ref=134_134521-x99q1iutux&title=Workforce-and-Safety-in-Long-Term-Care-during-the-COVID-19-pandemic&_ga=2.161262333.1980204992.1632834217-847912467.1632834217 (accessed September 30, 2021)

[8] Giraud O Petiau A Rist B Touahria-Gaillard A Trenta A. ≪ Ça fait des années qu'on est confinés ≫. La crise sanitaire du Covid-19 révélatrice de la condition des proches aidant·e·s de personnes en situation de dépendance. Revue Francaise des Affaires Sociales. (2020) 243–60. 10.3917/rfas.204.0243

[9] Vandentorren S Bretin P Zeghnoun A Mandereau-Bruno L Croisier A Cochet C. August 2003 heat wave in france: risk factors for death of elderly people living at home. Eur J Public Health. (2006) 16:583–91. 10.1093/eurpub/ckl063 17028103

[10] Ogg J. HEATWAVE: Implications of the 2003 French Heat Wave for the Social Care of Older People. [Young Foundation Working Paper]. Paris: The Young Foundation (2005).

[11] Kucharski AJ Klepac P Conlan AJK Kissler SM Tang ML Fry H. Effectiveness of isolation, testing, contact tracing, and physical distancing on reducing transmission of SARS-CoV-2 in different settings: a mathematical modelling study. Lancet Infect Dis. (2020) 20:1151–60. 10.1101/2020.04.23.20077024 32559451 PMC7511527

[12] Block P Hoffman M Raabe IJ Dowd JB Rahal C Kashyap R. Social network-based distancing strategies to flatten the COVID-19 curve in a post-lockdown world. Nat Human Behav. (2020) 4:588–96. 10.1038/s41562-020-0898-6 32499576

[13] Campos-Castillo C. Gender divides in engagement with COVID-19 information on the internet among U.S. Older Adults. J Gerontol Series B. (2021) 76:e104–10. 10.1093/geronb/gbaa133 32845009 PMC7499743

[14] Martins Van Jaarsveld G. The effects of COVID-19 among the elderly population: a case for closing the digital divide. Front Psychiatry. (2020) 11:577427. 10.3389/fpsyt.2020.577427 33304283 PMC7693633

[15] Lambert A Cayouette-Remblière J Guéraut É Roux GL Bonvalet C Girard V. Neighbourliness during lockdown in France. Populat Soc. (2020) 578:1–4. 10.3917/popsoc.578.0001

[16] Wilson-Genderson M Heid AR Cartwright F Collins AL Pruchno R. Change in loneliness experienced by older men and women living alone and with others at the onset of the COVID-19 pandemic. Res Aging. (2022) 44:369–81. 10.1177/01640275211026649 34344251 PMC9039590

[17] Atzendorf J Gruber S. Depression and loneliness of older adults in Europe and Israel after the first wave of covid-19. Eur J Ageing. (2021) 1–13. 10.1007/s10433-021-00640-8 34456660 PMC8383247

[18] Buffel T Yarker S Phillipson C Lang L Lewis C Doran P. Locked down by inequality: older people and the COVID-19 pandemic. Urban Stud. (2021). 10.1177/00420980211041018. [Epub ahead of print]. PMC10230293 37273496

[19] García-Prado A González P Rebollo-Sanz YF. Lockdown strictness and mental health effects among older populations in Europe. Econ Human Biol. (2022) 45:101116. 10.1016/j.ehb.2022.101116 35193043

[20] Whitehead BR Torossian E. Older adults' experience of the COVID-19 pandemic: a mixed-methods analysis of stresses and joys. Gerontologist. (2021) 61:36–47. 10.1093/geront/gnaa126 32886764 PMC7499618

[21] Arpino B Pasqualini M Bordone V Solé-Auró A. Older people's nonphysical contacts and depression during the COVID-19 lockdown. Gerontologist. (2021) 61:294. 10.1093/geront/gnab014 32977334 PMC7543583

[22] Green MJ Whitley E Niedzwiedz CL Shaw RJ Katikireddi SV. Social contact and inequalities in depressive symptoms and loneliness among older adults: a mediation analysis of the English Longitudinal Study of Ageing. SSM - Population Health. (2021) 13:100726. 10.1016/j.ssmph.2021.100726 33521227 PMC7820553

[23] Davies K Maharani A Chandola T Todd C Pendleton N. The longitudinal relationship between loneliness, social isolation, and frailty in older adults in England: a prospective analysis. Lancet Healthy Longevity. (2021) 2:e70–77. 10.1016/S2666-7568(20)30038-6 36098160

[24] Berg RL Cassells JS. Second Fifty Years: Promoting Health Preventing Disability. Washington, DC: National Academies Press (1992). Available online at: https://public.ebookcentral.proquest.com/choice/publicfullrecord.aspx?p=3376581 (accessed March 12, 2022) 25144081

[25] Warszawski J Beaumont A-L Seng R de Lamballerie X Rahib D Lydié N. Prevalence of SARS-Cov-2 antibodies and living conditions: the French national random population-based EPICOV cohort. BMC Infect Dis. (2022) 22:41. 10.1186/s12879-021-06973-0 35000580 PMC8743062

[26] Milner A Jumbe S. Using the right words to address racial disparities in COVID-19. Lancet Public Health. (2020) 5:e419–20. 10.1016/S2468-2667(20)30162-6 32707127 PMC7373398

[27] Schröder-Butterfill E Marianti R. A framework for understanding old-age vulnerabilities. Ageing Soc. (2006) 26:9–35. 10.1017/S0144686X05004423 23750062 PMC3672844

[28] Vignoli D Tanturri ML Acciai F. Home bitter home? Gender, living arrangements, and the exclusion from homeownership among older Europeans. Genus. (2016) 72:9. 10.1186/s41118-016-0014-y 28035141 PMC5153721

[29] Gaymu J Springer S. Living conditions and life satisfaction of older Europeans living alone: a gender and cross-country analysis. Ageing Soc. (2010) 30:1153–75. 10.1017/S0144686X10000231

[30] Delbès C Gaymu J. The shock of widowhood on the eve of old age: male and female experiences. Population. (2002) 57:885–914. 10.3917/pope.206.0885

[31] Fabisiak B Jankowska A Kłos R. Attitudes of polish seniors toward the use of public space during the first wave of the COVID-19 pandemic. Int J Environ Res Public Health. (2020) 17:8885. 10.3390/ijerph17238885 33260396 PMC7729857

[32] Galasso V Pons V Profeta P Becher M Brouard S Foucault M. Gender differences in COVID-19 attitudes and behavior: Panel evidence from eight countries. Proc Natl Acad Sci USA. (2020) 117:27285–91. 10.3386/w27359 33060298 PMC7959517

[33] Rattay P Michalski N Domanska OM Kaltwasser A Bock FD Wieler LH. Differences in risk perception, knowledge and protective behaviour regarding COVID-19 by education level among women and men in Germany. results from the COVID-19 Snapshot Monitoring. (COSMO) study. PLoS One. (2021) 16:e0251694. 10.1371/journal.pone.0251694 33979413 PMC8116045

[34] Schehl B Leukel J Sugumaran V. Understanding differentiated internet use in older adults: a study of informational, social, and instrumental online activities. Comput Human Behav. (2019) 97:222–30. 10.1016/j.chb.2019.03.031

[35] Wolf MS Feinglass J Thompson J Baker DW. In search of ‘low health literacy’: threshold vs. gradient effect of literacy on health status and mortality. Soc Sci Med. (2010) 70:1335–41. 10.1016/j.socscimed.2009.12.013 20167411

[36] Gilleard C Higgs P. Internet use and the digital divide in the English longitudinal study of ageing. Eur J Ageing. (2008) 5:233. 10.1007/s10433-008-0083-7 28798575 PMC5546327

[37] European Parliamentary Research Service. Older People in the European Union's Rural Areas: Issues Challenges : In Depth Analysis. LU: Publications Office. (2020). 36 p. Available online at: https://data.europa.eu/doi/10.2861/114962 (accessed February 26, 2021)

[38] Heylen L Mortelmans D Hermans M Boudiny K. The intermediate effect of geographic proximity on intergenerational support: a comparison of France and Bulgaria. DemRes. (2012) 27:455–86. 10.4054/DemRes.2012.27.17

[39] Martineau A Plard M. Are elderly immigrants meeting the challenges of successful aging? Review of literature on the aging of elderly migrants in France. Cybergeo Eur J Geography. 10.4000/cybergeo.33224

[40] DiMaggio P Hargittai E Celeste C Shafer S. From unequal access to differentiated use: a literature review and agenda for research on digital inequality. In: Inequality in the United States: A Reader. New York, NT: Routledge (2002). p. 73.

[41] Mitchell UA Chebli PG Ruggiero L Muramatsu N. The digital divide in health-related technology use: the significance of race/ethnicity. Gerontologist. (2019) 59:6–14. 10.1093/geront/gny138 30452660

