# Supplementary material for: Social Isolation Among Older Adults in the Time of COVID-19: A Gender Perspective
Source: Front Public Health. 2022 Jun 9;10:840940. doi: 10.3389/fpubh.2022.840940 (PMC9228032; doi:10.3389/fpubh.2022.840940)
Supplement: Supplementary file 1 [file Table_1.pdf]

	Women (%)						Men (%)					
	**65-69**	**70-74**	**75-79**	**80-84**	**85 +**	**Total**	**65-69**	**70-74**	**75-79**	**80-84**	**85 +**	**Total**
**Total n (%)**	4221 (27.6)	3449 (25.2)	1654 (16.8)	1025 (15.6)	843 (14.7)	11192 (100)	3806 (30)	3311 (28.3)	1647 (17)	996 (14.7)	591 (10)	10351 (100)
----------**Socio-professional category combined with level of qualification:**												
Farmers, self-employed and entrepreneurs	6.8	9.7	11.9	15.4	17.9	11.4	12.3	14.3	17.9	20.6	22.5	16.1
Senior executives professionals	14	13.8	13.3	10	7.8	12.3	25.2	29.2	30.8	29.5	24.9	27.9
Middle executive professionals	19	18.5	16.5	13.2	9.7	16.2	21.8	20.8	17.8	19.7	18	20.1
Skilled employees and skilled manual workers	8.3	7.2	5.6	6.5	7.9	7.2	20.1	18.1	16.1	13.2	16.5	17.5
Unskilled employees and unskilled manual workers	42.3	41.6	42.2	39	33.6	40.3	17.4	15.1	15	15.6	16.3	16
Never worked and others	9.5	9.3	10.4	15.9	23.1	12.6	3.2	2.5	2.4	1.4	1.8	2.5
----------**Formal education:**												
No diploma	12.7	13.9	16.9	22	28	17.4	11.1	12.8	15.4	15.4	23.1	14.1
Primary education	25.8	32.2	32.6	39.2	45.8	33.6	17.4	17.7	21.4	26.9	32.2	21.1
Vocational secondary	24.5	22.5	19.6	16.4	9.1	19.6	32.6	30.7	27.2	24.5	19	28.6
High school	15.7	13.6	15.5	12.2	7.4	13.4	14.2	14.2	13.8	13.8	11	13.8
High school + 2 to 4 years	17.2	14.4	11.6	7.9	8.2	12.7	16.2	15.1	11.7	9.4	5.8	13.1
High school + 5 or more years	4.1	3.5	3.8	2.3	1.5	3.2	8.5	9.5	10.5	9.9	8.9	9.4
----------**Perceived financial situation:**												
Comfortable	12.3	14.2	13.1	14	12.9	13.3	14.1	16.4	17.7	21	19	16.9
Decent	44.7	47.1	46.9	48.7	44.5	46.2	46.2	47.6	46.4	47.2	48.1	47
Just enough	35.2	32.6	35	32.4	35.5	34.1	32.7	29.7	32	28.6	28.3	30.7
Difficult to impossible without going into debt	7.8	6.1	5	4.9	7.1	6.3	7	6.2	3.9	3.2	4.6	5.5
----------**Populations size of municipality:**												
Rural area	24.9	22	25.7	22.5	20	23.2	28.8	25.1	24.8	23.7	24.8	25.9
<50 000 inhabitants	28.2	27.8	25.9	28.5	26.8	27.6	28.1	28.4	27.1	31.9	25.3	28.3
[50 000-200 000] inhabitants	12.3	13.1	13.7	14.1	12.5	13	10.9	12	14.6	12.8	11.9	12.2
>200 000 inhabitants	22.8	25	22.8	22.9	27.1	24	21.6	22.9	21.6	21.6	22.6	22
Paris	11.8	12.2	11.9	12.1	13.6	12.2	10.6	11.6	11.9	10.1	15.4	11.5
----------**Household composition:**												
Living alone	27.7	30.3	38.9	47.4	63.4	38.6	17.6	16.2	14.8	16.9	30.3	17.9

With a partner and with or without children	62.5	61.7	52.6	41.9	18.7	51		74.2	77.4	79.3	77.5	60.5	75.1
Other compositions	9.8	8.1	8.6	10.7	17.8	10.5		8.2	6.4	5.8	5.6	9.2	7
**----------Ethno-racial status:**													
Mainstream population	83.9	84.5	82.3	84.1	82.2	83.5		83	81.7	81.5	82.7	80.6	82.1
Racialized first or second-generation immigrants and DOM descendants	5.4	4.1	3	3.9	2.4	4		6.4	6.2	6.2	5.4	4.5	6
Non-racialized first or second-generation immigrants	10.7	11.4	14.7	12	15.4	12.5		10.6	12.1	12.3	11.9	14.9	11.9
**----------Perceived health status:**													
Very good	19.9	16.5	10.9	8.5	6.9	13.8		18.3	15.3	13.4	9.8	8.4	14.4
Good	48.7	47.6	47	40.5	29	44		48.7	48.3	44	42.3	37.3	45.7
Fair	27	30.4	34.4	40.9	49	34.5		26.6	30.2	34.3	38.3	40.6	32
Bad to very bad	4.5	5.5	7.7	10	15.1	7.7		6.4	6.2	8.3	9.6	13.7	7.8
**----------Declared chronic disease or physical limitation:**													
Did not declare any	43.8	39.7	36.5	28.1	20	35.6		39.9	36.4	31.5	27.2	24.8	34.1
Declared at least one	56.2	60.3	63.5	71.9	80	64.4		60.1	63.6	68.5	72.8	75.2	65.9
**----------Declared chronic anxiety or depression:**													
Declared chronic anxiety or depression	8.3	9.2	10.8	12.6	11.1	10.1		3.7	2.9	3.9	4.4	5.6	3.8
Did not declare chronic anxiety or depression	91.7	90.8	89.2	87.4	88.9	89.9		96.3	97.1	96.1	95.6	94.4	96.2
**----------Average alcohol consumption:**													
Every day	8	8.5	11	11	10	9.4		22.5	25.8	30.9	36.9	33.8	28.1
Once to several times per week	19	18.5	15.8	14.4	9.1	16.2		28.2	25.5	22.2	20.9	16.8	24.2
Once to several times per month	17.8	16.6	14.1	11.8	9.8	14.8		17.2	16.7	14.1	11	9.1	14.8
Less often	18	17.7	17.1	14.6	13.6	16.6		12.2	12	12.7	11.7	13.7	12.3
Never	37.2	38.6	42	48.2	57.5	43.1		19.9	20	20.1	19.5	26.5	20.6
**----------Average Internet use in the past 3 months:**													
Regularly	81.6	75.9	60.8	44.5	23.8	62.3		86.1	81.5	73.3	61.2	39.2	74.3
Occasionally	5.1	5.4	6.5	6.2	4.2	5.4		3.9	3.5	4.4	5.7	5.3	4.3
No usage	13.3	18.6	32.5	49.1	71.8	32.1		9.9	15	22.3	33.1	55.5	21.4
**----------How many times the person went out in the last 7 days:**													
More than 6 times	15.7	15.3	12.3	11.8	6.5	13.1		23.6	21.5	20.1	17.7	13.1	20.5
2 to 5 times	45.6	45.1	41.3	38.1	26.2	40.7		46.4	45.4	43.6	43.8	33.2	43.9

Only once	29.5	28.7	30	26.1	19.1	27.4	23.9	24.4	24.1	23	21.2	23.7
None	9.1	10.9	16.4	24	48.1	18.9	6	8.8	12.1	15.6	32.6	11.9
**----------Date of questionnaire:**												
02/05-10/05	42.5	42.5	40.7	41.8	39.1	41.6	46	44.8	42.6	39.9	36.5	43.2
11/05-17/05	30.6	29.2	29.9	25.9	26.7	28.8	27.2	27.7	29.8	27	31	28.1
18/05-01/06	26.8	28.3	29.4	32.3	34.2	29.6	26.9	27.5	27.6	33	32.5	28.6

Notes: N = 21.543
12.6% of older women perceive their financial situation as “comfortable” (11.8% for women aged 65 to 69 and 13% for women aged 80 to 84)

Supplementary Table 1. Characteristics of Sample Respondents by Age Group and Gender

Total n (%)	Women<br>11,192 (55.9)	Men<br>10,351 (44.1)
**----------Age:**		
65-69	53.9	46.1
70-74	53.1	46.9
75-79	55.7	44.3
80-84	57.4	42.6
85+	65.3	34.7
**----------Socio-professional category combined with level of qualification:**		
Farmers. self-employed and entrepreneurs	47.3	52.7
Senior executives professionals	35.9	64.1
Middle executive professionals	50.5	49.5
Skilled employees and skilled manual workers	34.5	65.5
Unskilled employees and unskilled manual workers	76.2	23.8
Never worked and others	86.7	13.3
**----------Formal education:**		
No diploma	61	39
Primary education	66.9	33.1
Vocational secondary	46.6	53.4
High school	55.2	44.8
High school + 2 to 4 years	55.3	44.7
High school + 5 or more years	30.6	69.4
**----------Perceived financial situation:**		
Comfortable	50	50
Decent	55.6	44.4
Just enough	58.5	41.5
Difficult to impossible without going into debt	59.5	40.5
**----------Populations size of municipality:**		
Rural area	53.2	46.8
<50 000 inhabitants	55.3	44.7
[50 000-200 000] inhabitants	57.5	42.5
>200 000 inhabitants	58	42
Paris	57.4	42.6
**----------Household composition:**		
Living alone	73.2	26.8
With a partner and with or without children	46.3	53.7
Other compositions	65.5	34.5
**----------Ethno-racial status:**		
Mainstream population	56.4	43.6
Racialized first or second-generation immigrants and DOM descendants	45.8	54.2
Non-racialized first or second-generation immigrants	57	43
**----------Perceived health status:**		
Very good	54.9	45.1
Good	55	45
Fair	57.8	42.2
Bad to very bad	55.5	44.5
**----------Declared chronic disease or physical limitation:**		
Did not declare any	56.9	43.1
Declared at least one	55.4	44.6

----------**Declared chronic anxiety or depression:**		
Declared chronic anxiety or depression	77.1	22.9
Did not declare chronic anxiety or depression	54.3	45.7
----------**Average alcohol consumption:**		
Every day	29.8	70.2
Once to several times per week	45.9	54.1
Once to several times per month	55.9	44.1
Less often	63.1	36.9
Never	72.7	27.3
----------**Average Internet use in the past 3 months:**		
Regularly	51.6	48.4
Occasionally	61.8	38.2
No usage	65.5	34.5
----------**How many times the person went out in the last 7 days:**		
More than 6 times	44.7	55.3
2 to 5 times	54.1	45.9
Only once	59.5	40.5
None	66.8	33.2

Notes: N = 21.543

65.3% of individuals 85+ were women

Supplementary Table 2. Characteristics of Sample Respondents by Gender

	Lives alone	Did not go out in the last 7 days	Does not use the Internet
	aOR [95% CI]	aOR [95% CI]	aOR [95% CI]
**----------Sex:**			
Men (ref)	1	1	1
Women	**2.72 [2.53 ; 2.92]**	**1.53 [1.39 ; 1.68]**	**1.30 [1.20 ; 1.44]**
**----------Age:**			
65-69 (ref)	1	1	1
70-74	1.05 [0.96 ; 1.14]	**1.30 [1.14 ; 1.47]**	**1.54 [1.37 ; 1.74]**
75-79	**1.35 [1.22 ; 1.49]**	**1.96 [1.71 ; 2.26]**	**2.93 [2.57 ; 3.34]**
80-84	**1.85 [1.65 ; 2.08]**	**2.77 [2.38 ; 3.22]**	**5.69 [4.95 ; 6.54]**
85 +	**3.83 [3.37 ; 4.35]**	**7.57 [6.48 ; 8.83]**	**13.34 [11.41 ; 15.60]**
**----------Formal education:**			
No diploma	**0.74 [0.64 ; 0.88]**	**2.17 [1.82 ; 2.58]**	**10.61 [8.91 ; 12.63]**
Primary education	0.90 [0.81 ; 1.01]	**1.61 [1.38 ; 1.88]**	**3.94 [3.36 ; 4.62]**
Vocational secondary	**0.79 [0.71 ; 0.88]**	**1.34 [1.15 ; 1.56]**	**2.46 [2.09 ; 2.90]**
High school (ref)	**1**	1	1
High school + 2 to 4 years	**1.03 [0.92 ; 1.15]**	0.87 [0.73 ; 1.05]	0.93 [0.76 ; 1.14]
High school + 5 or more years	**0.98 [0.85 ; 1.13]**	0.97 [0.78 ; 1.21]	**0.49 [0.36 ; 0.66]**
**----------Perceived financial situation:**			
Comfortable (ref)	1	1	1
Decent	**1.18 [1.07 ; 1.31]**	1.06 [0.93 ; 1.22]	1.10 [0.96 ; 1.26]
Just enough	**1.73 [1.56 ; 1.93]**	**1.18 [1.02 ; 1.37]**	**1.48 [1.28 ; 1.72]**
Difficult to impossible without going into debt	**3.13 [2.65 ; 3.70]**	1.17 [0.93 ; 1.48]	**1.52 [1.23 ; 1.89]**
**----------Population size of municipality:**			
Rural area	**0.75 [0.67 ; 0.84]**	**1.54 [1.31 ; 1.80]**	**1.44 [1.24 ; 1.67]**
<50 000 inhabitants	**0.88 [0.79 ; 0.98]**	**1.17 [1.00 ; 1.37]**	**1.27 [1.09 ; 1.47]**
[50 000-200 000] inhabitants (ref)	1	1	1
>200 000 inhabitants	1.03 [0.92 ; 1.15]	1.16 [0.99 ; 1.37]	1.07 [0.91 ; 1.24]
Paris	1.08 [0.95 ; 1.24]	0.96 [0.78 ; 1.16]	0.89 [0.74 ; 1.08]
**----------Household composition:**			
Living alone (ref)		1	1
With a partner and with or without children		**1.12 [1.00 ; 1.25]**	**0.71 [0.64 ; 0.79]**

Other compositions		**1.75 [1.48 ; 2.06]**	1.02 [0.87 ; 1.20]
**----------Ethno-racial status:**			
Mainstream population (ref)	1	1	1
Racialized first or second-generation immigrants and DOM descendants	**0.69 [0.56 ; 0.83]**	**1.85 [1.50 ; 2.29]**	**1.43 [1.16 ; 1.77]**
Non-racialized first or second-generation immigrants	1.01 [0.91 ; 1.12]	1.05 [0.91 ; 1.21]	1.08 [0.95 ; 1.24]
**----------Perceived health status:**			
Very good (ref)	1	1	1
Good	0.98 [0.89 ; 1.08]	1.13 [0.97 ; 1.32]	**1.34 [1.16 ; 1.56]**
Fair	1.04 [0.94 ; 1.15]	**1.77 [1.51 ; 2.06]**	**1.93 [1.66 ; 2.25]**
Bad to very bad	1.09 [0.93 ; 1.28]	**5.10 [4.22 ; 6.15]**	**3.05 [2.51 ; 3.70]**
**----------Date of questionnaire:**			
02/05-10/05		1	
11/05-17/05		**0.82 [0.74 ; 0.91]**	
18/05-01/06		**0.42 [0.37 ; 0.47]**	

Notes: N = 21.543, aOR = adjusted odd ratio, significant associations are indicated in bold

Supplementary Table 3. Logistic Regressions of Living Alone, Not Having Gone Out in the Past Week and Never Using the Internet

	Did not go out in the last 7 days		Does not use the Internet	
	**Lives alone**	**Does not live alone**	**Lives Alone**	**Does not live alone**
----------**Sex:**				
Men (ref)	1	1	1	1
Women	**1.54 [1.24 ; 1.93]**	**1.59 [1.43 ; 1.76]**	**1.25 [1.04 ; 1.51]**	**1.39 [1.25 ; 1.54]**
----------**Age:**				
65-69 (ref)	1	1	1	1
70-74	1.07 [0.79 ; 1.45]	**1.34 [1.17 ; 1.54]**	**1.40 [1.09 ; 1.80]**	**1.58 [1.38 ; 1.82]**
75-79	**1.68 [1.23 ; 2.30]**	**2.04 [1.75 ; 2.39]**	**2.82 [2.18 ; 3.66]**	**2.97 [2.54 ; 3.46]**
80-84	**2.65 [1.94 ; 3.62]**	**2.81 [2.35 ; 3.34]**	**5.42 [4.16 ; 7.06]**	**5.86 [4.97 ; 6.92]**
85 +	**8.02 [6.06 ; 10.62]**	**7.37 [6.07 ; 8.94]**	**13.89 [10.68 ; 18.05]**	**13.37 [10.94 ; 16.34]**
----------**Formal education:**				
No diploma	**2.41 [1.71 ; 3.40]**	**2.11 [1.73 ; 2.58]**	**9.70 [7.09 ; 13.28]**	**11.23 [9.08 ; 13.88]**
Primary education	**1.64 [1.20 ; 2.23]**	**1.59 [1.33 ; 1.90]**	**3.57 [2.72 ; 4.69]**	**4.14 [3.40 ; 5.04]**
Vocational secondary	**1.58 [1.14 ; 2.18]**	**1.27 [1.06 ; 1.51]**	**2.12 [1.58 ; 2.82]**	**2.63 [2.15 ; 3.21]**
High school (ref)	1	1	1	1
High school + 2 to 4 years	0.83 [0.57 ; 1.22]	0.88 [0.72 ; 1.08]	0.84 [0.59 ; 1.19]	0.99 [0.77 ; 1.27]
High school + 5 or more years	0.96 [0.58 ; 1.60]	0.97 [0.76 ; 1.23]	**0.49 [0.28 ; 0.86]**	**0.49 [0.34 ; 0.71]**
----------**Perceived financial situation:**				
Comfortable (ref)	1	1	1	1
Decent	1.09 [0.81 ; 1.47]	1.08 [0.92 ; 1.25]	0.95 [0.73 ; 1.23]	1.17 [1.00 ; 1.39]
Just enough	1.25 [0.92 ; 1.71]	**1.20 [1.01 ; 1.42]**	1.36 [1.03 ; 1.78]	**1.57 [1.32 ; 1.88]**
Difficult to impossible without going into debt	1.11 [0.73 ; 1.69]	**1.35 [1.02 ; 1.79]**	1.25 [0.87 ; 1.81]	**1.82 [1.39 ; 2.38]**
----------**Population size of municipality:**				
Rural area	**1.52 [1.11 ; 2.08]**	**1.55 [1.29 ; 1.87]**	**1.89 [1.43 ; 2.50]**	**1.30 [1.09 ; 1.54]**
<50 000 inhabitants	1.07 [0.79 ; 1.46]	1.19 [0.99 ; 1.44]	**1.61 [1.23 ; 2.10]**	1.14 [0.96 ; 1.36]
[50 000-200 000] inhabitants (ref)	1	1	1	1
>200 000 inhabitants	1.16 [0.85 ; 1.59]	1.17 [0.96 ; 1.42]	1.13 [0.85 ; 1.49]	1.04 [0.87 ; 1.25]
Paris	0.74 [0.50 ; 1.09]	1.07 [0.85 ; 1.35]	0.91 [0.65 ; 1.28]	0.91 [0.72 ; 1.14]
----------**Ethno-racial status:**				
Mainstream population (ref)	1	1	1	1
Racialized first or second-generation immigrants and DOM descendants	1.51 [0.91 ; 2.48]	**2.06 [1.63 ; 2.60]**	**1.61 [1.04 ; 2.49]**	**1.43 [1.12 ; 1.81]**

Non-racialized first or second-generation immigrants	1.06 [0.80 ; 1.39]	1.06 [0.90 ; 1.25]	1.00 [0.78 ; 1.28]	1.12 [0.96 ; 1.31]
**----------Perceived health status:**				
Very good (ref)	1	1	1	1
Good	**1.47 [1.02 ; 2.10]**	1.07 [0.90 ; 1.26]	1.27 [0.97 ; 1.68]	**1.36 [1.14 ; 1.62]**
Fair	**2.50 [1.75 ; 3.57]**	**1.61 [1.35 ; 1.92]**	**1.92 [1.46 ; 2.53]**	**1.93 [1.61 ; 2.31]**
Bad to very bad	**7.15 [4.73 ; 10.81]**	**4.72 [3.81 ; 5.84]**	**2.64 [1.83 ; 3.80]**	**3.21 [2.55 ; 4.04]**
**----------Date of questionnaire:**				
02/05-10/05	1	1		
11/05-17/05	0.88 [0.71 ; 1.09]	**0.81 [0.72 ; 0.91]**		
18/05-01/06	**0.52 [0.41 ; 0.65]**	**0.39 [0.33 ; 0.45]**		

Notes: N = 21.543

Supplementary Table 4. Logistic Regressions of Not Having Gone Out in the Past Week and Never Using the Internet, by household composition (Lives alone yes/no)
